# Impact of salpingectomy on ovarian response to hormonal stimulation in in vitro fertilization (IVF) cycles

**DOI:** 10.1186/s13048-026-02114-w

**Published:** 2026-05-22

**Authors:** Yohan Benchimol, Stéphane Sanchez, Catherine Racowsky, Rossana Cicinelli, Achraf Benammar, Jean-Marc Ayoubi

**Affiliations:** 1 Department of Obstetrics, Gynecology and Reproductive Medicine, Hospital Foch, Suresnes, 92150 France; 2 University Versailles, UMR BREED 1198, INRAE -Paris Saclay Université- UVSQ - ENVA, Jouy-en-Josas, France

**Keywords:** Salpingectomy, In vitro fertilization, Ovarian response, Follicular recruitment

## Abstract

**Background:**

The coagulation-section of the mesosalpinx during the salpingectomy can lead to injuries on the ovarian vascularization, and thus to the ovarian response to hormonal stimulation. Does salpingectomy affect negatively the follicles recruitment in in vitro fertilization (IVF) cycles? We conducted a monocentric retrospective case-control study including all patients undergoing IVF cycle with a history of unilateral salpingectomy for hydrosalpinx (HSX) or ectopic pregnancy (EP) (Group S), and each of them was matched to two controls (Group C) treated in the same month, according to their: age, AMH level, and BMI. The difference in follicle recruitment the day of trigger between the two ovaries of each patient was calculated. The primary endpoint was the difference between the two groups in the total number of follicles with a diameter ≥ 13 mm. Secondary endpoints were the difference between groups regarding the number of (1) mature follicles (diameter ≥ 16 mm) and intermediate follicles [13.0–15.5 mm]; (2) cycle cancellation rate; and (3) total number of oocytes and mature oocytes retrieved. The same comparisons were also carried out on subgroups according to the indication for salpingectomy (HSX or EP).

**Results:**

114 and 228 patients were included in Groups S and C, respectively. Compared to Group C, Group S patients showed a greater difference between ovaries in the total number of follicles recruited (0.76 ± 3.95 vs. -0.18 ± 3.37, *p* = 0.002) and in the number of mature follicles (0.51 ± 2.91 vs. -0.14 ± 2.85, *p* = 0.05). No significant differences were observed for the number of intermediate follicles, or the total number of oocytes or mature oocytes retrieved. However, the cycle cancellation rate was significantly higher in Group S (13.2% vs. 3.1%, *p* < 0.001). Subgroup analysis revealed no significant differences between each of the Group S subgroups (HSX and EP) regarding follicular recruitment. Nevertheless, both subgroups had significantly more cancelled cycles compared with their respective controls (HSX: 12.5% vs. 4.4%, *p* < 0.001; EP: 8.8% vs. 1.8%, *p* = 0.004).

**Conclusion:**

Salpingectomy has a negative impact on follicles recruitement in IVF cycles. This risk must be taken into consideration when scheduling this surgery.

## Background

A recent study has estimated that one in five couples suffer from infertility [1], many of whom resort to assisted reproductive technologies (ART) for family-building. In vitro fertilization (IVF) has been a real revolution for couples wishing to procreate. Initially developed to treat infertility of tubal origin (obstruction, absence or alteration of the Fallopian tubes), which represents between 30% [2] and 67% [3] of infertility diagnoses, its indications have expanded over time to treat different female and male infertility etiologies.

One of the major causes of the absence of Fallopian tubes is from their removal due to an ectopic pregnancy (EP) with ruptured tubes, or from an EP that cannot be treated with methotrexate, thus resulting in secondary tubal infertility [4]. Paradoxically, planned Fallopian tube removal, also called salpingectomy, has been shown to improve fertility in cases of hydrosalpinx (HSX) [5], which has been associated with accumulation of intrauterine fluid rich in embryo and gameto-toxic inflammatory cytokines, as well as possible disruption of implantation caused by a mechanical effect [6].

Despite salpingectomy being performed frequently in routine gynecology and not typically being a complex procedure [7], the coagulation-section of the mesosalpinx during the intervention can lead to vascular injuries. As the tubes and the ovaries have a common vascularization, cutting and coagulating the lateral and medial tubal artery during the surgery can have an impact on the ovarian vascularization.

Based on this tubal-ovarian physiopathology, many studies have focused on the impact of salpingectomy on ovarian function in terms of: (1) ovarian reserve, reflected by antral follicle count (AFC) [8–13], basal follicle stimulating hormone (FSH) [8–11, 13–15], or antimüllerian hormone (AMH) levels [8, 9, 11–13]; (2) ovarian response in the context of controlled ovarian stimulation (COS): total dose of used gonadotropins, total length of COS [10, 14], number of retrieved oocytes [9–11, 14–16], or obtained embryos [9, 16]; and (3) pregnancy or live-birth rates [9, 10, 15, 16]. Nevertheless, the results of many of these studies remain contradictory, due to methodological biases (e.g. comparison of young fertile patients with either infertile women undergoing IVF or post-menopausal women, or comparison of non-homogeneous groups).

In a previous study, our team focused on the ovarian response after salpingectomy during IVF stimulation in terms of number of mature follicles (diameter ≥ 16 mm) on the day of trigger [17] and, to avoid the biases of previous studies, we chose to evaluate the impact of unilateral salpingectomy by comparing the response between ovaries in the same patient before and after salpingectomy. Our findings suggested that salpingectomy negatively impacts recruitment of mature follicles. However, this study had the weakness of being carried out on only 28 patients, as it was rare to find patients who had undergone IVF, then unilateral salpingectomy and another IVF cycle in our center. To address this weakness, we performed the present study, the aim of which was to confirm or refute our previous results on a larger cohort. In this investigation, we evaluated the impact of salpingectomy on ovarian response in terms of follicular recruitment on the day of trigger, cycle cancellation rate and oocyte yield by comparing two homogeneous populations.

## Methods

### Study populations

This retrospective matched case-control study was conducted using data from a single university affiliated center of ART at Foch Hospital, Suresnes, France. All patients 18–43 years undergoing an IVF cycle between September 2016 and April 2024 with a history of unilateral salpingectomy were included as the salpingectomy group (Group S). Each patient in this group was matched to two control patients (Group C) undergoing COS for IVF in the same month and year, according to their: age (< 30, [30-33.9], [34-35.9], [36-37.9], [38-39.9], [40-40.9], [41- 41.9], ≥ 42 years old), body mass index (BMI) (< 18, [18-24.9], [25- 29.9], [30- 34.9], [35-39.9] and ≥ 40 kg/m2) and AMH levels (matched to equivalent values ± 0.1 ng/ml).

The exclusion criteria for both groups were history of any ovarian surgery (endometrioma, cysts etc.), partial or total oophorectomy or previous gonadotoxic treatment.

In Group S, the ovary on the side with an intact Fallopian tube was considered as the control ovary and that on the salpingectomy side was considered as the ipsilateral ovary.

### Ovarian stimulation

Patients received an antagonist protocol and began their COS with daily injections of gonadotropins: urinary FSH, recombinant FSH or menotropins. The initial dose, from 125 to 600 IU/day was started and was adjusted according to the ovarian response. Growth of ovarian follicles was assessed by hormonal blood levels and by measuring the mean diameter of each follicle (average measure between the two longest diameters) using a transvaginal ultrasonography high-frequency probe (Voluson S8 system, GE Healthcare, Chicago, IL, USA). On day 6, daily injections of a gonadotropin releasing hormone (GnRH) antagonist (Cétrotide^®^ 0.25, Merck Europe, Amsterdam, Holland) were introduced. Ovulation triggering by a human chorionic gonadotropin (hCG, Ovitrelle^®^ 250 µg, Merck Europe, Amsterdam, Holland) or GnRH-agonist (Décapeptyl^®^ 0.1 mg x 2, Ipsen Pharma, Boulogne-Billancourt, France) or both was achieved when at least 3 follicles measuring more than 17 mm in diameter were observed with a concordant estradiol (E2) level of > 200 pg/ml per mature follicle. Oocyte retrieval was performed 36 h later. If the hormonal response was insufficient (total E2 < 500 pg/ml) and/or the total number of mature follicles was less than three, the IVF cycle was cancelled [18].

Oocytes were fertilized with the partner or donor sperm by conventional IVF (cIVF) or intracytoplasmic sperm injection (ICSI) as indicated by sperm parameters. Oocyte maturity (metaphase II [MII] oocytes) was defined by the presence of at least one polar body and was determined on day 0 for ICSI cycles, after oocyte denudation in hyaluronidase (40 UI/mL) (FUJIFILM Irvine scientific, USA) or at day 1 for cIVF cycles after mechanical oocyte denudation.

### Data collection

Demographic characteristics (including age, smoking status, infertility type (primary or secondary), the duration of infertility and etiology, the basal AFC and hormonal levels as well as cycles characteristics (including COS protocol, daily and total gonadotropins doses, hormonal levels, follicle numbers and size, number of oocytes retrieved and number of MII oocytes, fertilization technique) were recorded. For patients in Group S, the first IVF attempt after salpingectomy was selected, and laterality of salpingectomy and its etiology (EP or HSX) were recorded.

### Outcome measures

The primary endpoint was the difference in total (T) number of follicles ≥ 13 mm in diameter on the day of trigger between the two ovaries of each patient according to the 2019 ESHRE guidelines [19]. Secondary endpoints were the difference in recruitment of mature (M) follicles (mean diameter ≥ 16 mm); and in intermediate (I) follicles (mean diameter between 13.0 and 15.5 mm), the cycle cancellation rate (CCR), and the number of retrieved oocytes and those that were mature. Differences in follicle and oocyte numbers were calculated for only those patients who underwent oocyte retrieval.

The difference in follicle number between the ipsilateral (salpingectomy side) and control ovary (left or right) for each patient in Group S was determined and this was then compared with the difference in response between the two control ovaries of her two matched patients in Group C. Due to the absence of equivalent laterality in the ovaries of Group C patients, the same laterality was retained as for each matched Group S patient. Differences in follicles number were therefore expressed as negative or positive values for each patient.

The cycle cancellation rate (CCR) was defined as the number of cancelled cycles of all started cycles (cancelled cycles + cycles that resulted in oocyte retrieval).

### Statistical analysis

Quantitative variables were described using their mean and standard deviation and were compared using the Student’s t-test. Qualitative variables were described using percentages. and were compared using the chi-square test. Bonferonni corrections were applied to avoid alpha risk inflation for qualitative variables. For quantitative analyses, we used a mixed effects linear regression with the status (experimental or control) as fixed effects and pairing strata as random effect. For all analyses, the significance level was set *p* < 0.05. All analyses were performed using SPSS 27.0 (Chicago, Inc).

Due to the limited sample size (< 200 patients) and the low number of outcome events, multivariable regression and propensity score methods were not performed. These approaches require an adequate number of events per variable to ensure model stability and avoid overfitting. Applying such methods in the present dataset would have led to unreliable estimates and potential bias. Therefore, analyses were restricted to unadjusted and carefully interpreted comparisons.

## Results

### Patient demographics and cycle characteristics

A total of 114 and 228 patients who had started their IVF cycles were included in the groups S and C, respectively. The distributions of etiologies and laterality of salpingectomies were identical between the two subgroups of patients.

No statistically significant differences were observed between the Group S and Group C regarding age, BMI, AFC, basal hormonal levels (Follicle stimulating hormone (FSH) ; Luteinizing hormone (LH) and Estradiol (E2)), duration of infertility or tobacco consumption (Table 1). However, more patients in Group S had secondary infertility compared to the Group C (61.4% vs. 39.9%; *p* < 0.001) with fewer patients presenting with idiopathic infertility (0% vs. 11.4%) or male factor infertility (10.5% vs. 34.2%). Not surprisingly, more patients in Group S had tubal factor compared with those in Group C (79.8% vs. 3.1%) and more were diagnosed with endometriosis (30.7% vs. 21.9%). The incidence of all other etiologies for infertility were similar between groups.

**Table 1:** Population characteristics between unilateral salpingectomized patients (Group S) and the matched controls (Group C)

	Group S	Group C	*P*-value
Patients (*n*)	114	228	
Mean age (y)	35.83 ± 4.40	35.79 ± 4.54	0.95
BMI (kg/m2)	25.13 ± 4.50	25.02 ± 4.46	0.82
AFC	16.64 ± 16.29	16.57 ± 12.93	0.97
Basal AMH (ng/ml)	2.37 ± 2.83	2.52 ± 3.39	0.69
Basal FSH (IU/l)	7.47 ± 3.24	7.09 ± 2.90	0.29
Basal LH (IU/l)	5.30 ± 2.67	4.88 ± 2.64	0.19
Basal E2 (pg/ml)	59.20 ± 59.59	53.07 ± 45.17	0.33
Infertility length (y)	4.29 ± 2.70	4.85 ± 8.15	0.48
Etiology of salpingectomy			
Ectopic pregnancy n(%)	57 (50)		
Hydrosalpinx n(%)	57 (50)		
Laterality of salpingectomy			
Left side n(%)	57 (50)		
Right side n(%)	57 (50)		
Smoking status			0.28
Non smoker n(%)	86 (75.4)	180 (78.9)	
Ex-smoker n(%)	16 (14.0)	28 (12.3)	
Current smokers n(%)	10 (8.8)	20 (8.8)	
Missing data n(%)	2 (1.8)	0 (0)	
Infertility			<0.001
Primary infertility n(%)	44 (38.6)	137 (60.1)	
Secondary infertility n(%)	70 (61.4)	91 (39.9)	
Etiology of infertility			
Idiopathic n(%)	0 (0)	26 (11.4)	
Tubal factors n(%)	91 (79.8)	7 (3.1)	
Male factors n(%)	12 (10.5)	78 (34.2)	
Endometriosis n(%)	35 (30.7)	50 (21.9)	
Poor ovarian reserve n(%)	44 (38,6)	91 (39,9)	
Dysovulation as PCOS n(%)	12 (10.5)	24 (10,5)	
Uterine factors n(%)	1 (0.88)	3 (1.3)	

Values are means +/- SD or n(%)

*Abbreviations: Group S* Salpingectomy group (cases), *Group C* Control group, *BMI* Body mass index, *AFC* Antral follicle count, *AMH* Antimüllerian hormone, *FSH* Follicle stimulating hormone, *LH* Luteinizing hormone, *E2* Estradiol, *y* Years, *PCOS* Polycystic ovary syndrome

Comparison of ovarian stimulation characteristics between the two groups revealed no significant differences for any variable assessed including pre-treatment protocol, ovulation triggering method, number of stimulation days or total dose of used gonadotropins (Table 2). However, fewer cycles used ICSI in Group S compared with Group C (26.4% vs. 51.1%; *p* < 0.001).

**Table 2:** Comparison of ovarian stimulation characteristics between unilateral salpingectomized patients (Group S) and the matched controls (Group C)

	Group S	Group C	*P*-value
Fertilization technique			0.003
cIVF n(%)	81 (73.6)	108 (48.9)	
ICSI n(%)	29 (26.4)	113 (51.1)	
Pre-treatment			0.41
None n(%)	42 (36.8)	68 (29.8)	
Estrogens (Provames) n(%)	1 (0.9)	3 (1.3)	
COC pill n(%)	71 (62.3)	157 (68.9)	
Daily dose of gonadotropin (IU)	459 ± 144	460 ± 154	0.92
Total dose of gonadotropin (IU)	5152 ± 2039	5183 ± 2135	0.9
Days number of ovarian stimulation (d ± SD)	11.1 ± 2.0	11.1 ± 2.0	0.99
Ovulation trigger			0.13
None n(%)	9 (7.9)	0 (0)	
hCG n(%)	19 (16.7)	38 (16.7)	
GnRH-agonist n(%)	18 (15.8)	56 (24.6)	
Both hCG + GnRH-agonist n(%)	68 (59.6)	134 (58.8)	

*Abbreviations: Group S* Salpingectomy group (cases), *Group C* Control group, *cIVF* Conventional in vitro fertilization, *ICSI* Intracytoplasmic sperm injection, *COC* Combined oral contraceptive, *d* Days, *hCG* Human chorionic gonadotropin, *GnRH* Gonadotropin releasing hormone

### Outcome measures

**Fig. 1:**
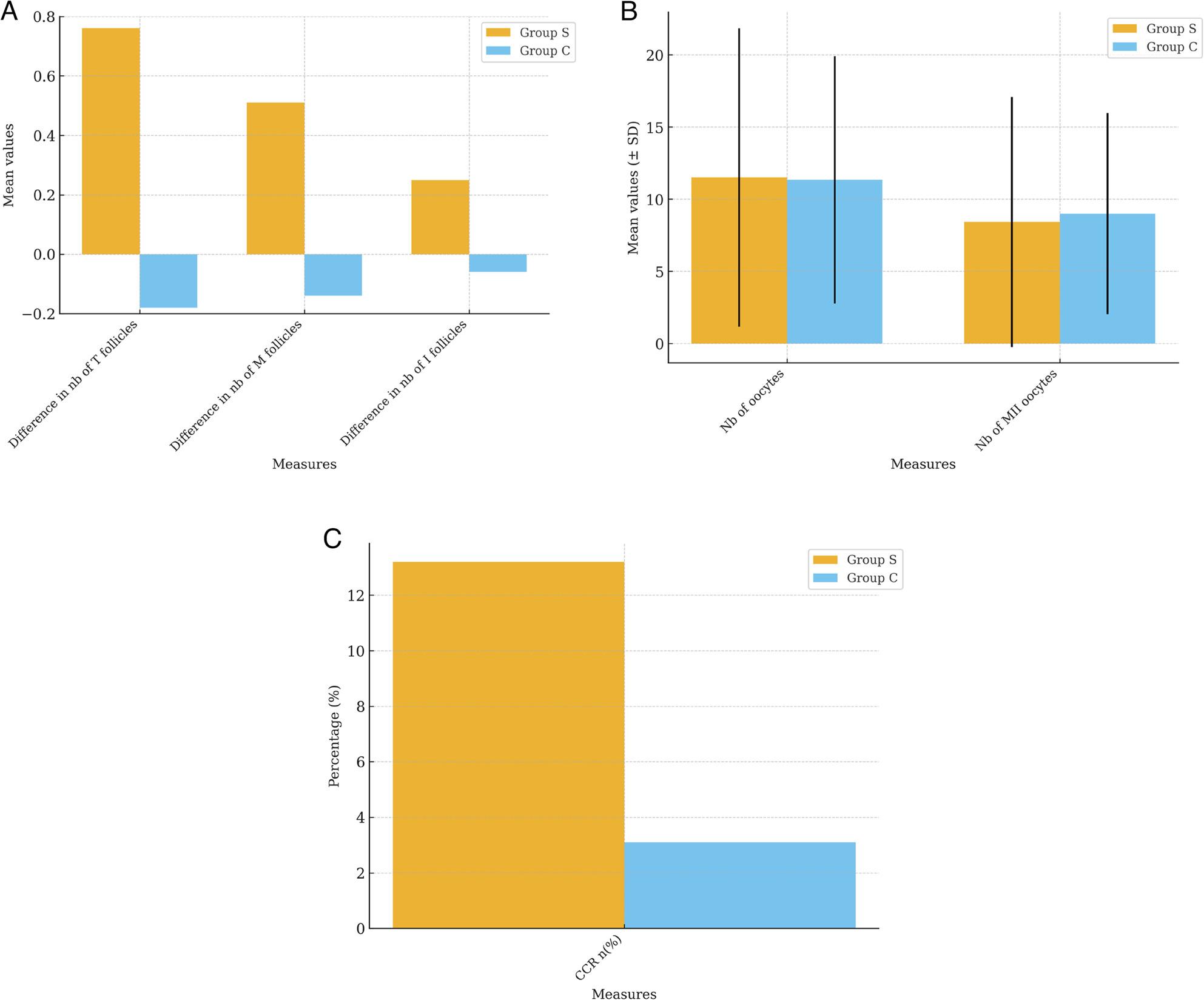
Assessment of ovarian stimulation and oocytes retrieval outcome in salpingectomy and control groups. **A** Comparison of recruited follicle difference between the two ovaries in patients from Group S and Group C. Difference refers to the mean difference between cases (Group S) and controls (Group C). *Abbreviations: Group S = salpingectomy group (cases) ; Group C= control group ; Nb = number ; T = total ; M = mature ; I = intermediate. ***B** Comparison of the number of retrieved and mature oocytes in patients from Group S and Group C. Mean value refers to the number of retrieved oocytes and those that were mature between between cases (Group S) and controls (Group C). Abbreviations: Group S = salpingectomy group (cases) ; Group C= control group ; Nb = number ; MII = metaphase II. **C** Comparison of IVF cycle cancellation rates in patients from Group S anf Group C. The cycle cancellation rate (CCR), in percentage, is defined as the number of cancelled cycles of all started cycles (cancelled cycles + cycles that resulted in oocyte retrieval). Abbreviations: Group S = salpingectomy group (cases) ; Group C= control group ; CCR = Cycle cancellation rate

Assessments of inter-ovarian response to COS within patients and overall differences between the two studied groups are shown in Table 3. In contrast to the slight mean difference in number of T recruited follicles identified between the two ovaries in Group C (-0.18 ± 3.37), this difference was statistically significantly greater for Group S, with more T follicles being recruited in the control ovary compared with the salpingectomy side (0.76 ± 3.95; *p* = 0.02). In contrast, no statistically significant inter-ovarian differences for either group were observed regarding the number of intermediate-size (I) follicles (0.25 ± 2.12 vs. -0.06 ± 2.08, Groups S and C, respectively; *p* = 0.19), or the number or M follicles (0.51 ± 2.91 vs. -0.14 ± 2.85; Groups S and C, respectively; *p* = 0.05), as shown in Fig. 1A. The differences in the total number of retrieved and mature oocytes were similar between the two groups (Fig. 1B). A statistically significant increase in CCR was observed in Group S compared to Group C (13.2% vs. 3.1% respectively, *p* < 0.001), also illustrated in Fig. 1C.

**Table 3:** Assessment of ovarian stimulation and oocytes retrieval outcomes in salpingectomy and control groups

	Group S	Group C	Difference	*P*-value	95% CI
Difference in nb of T follicles	0.76 ± 3.95	-0.18 ± 3.37	0.94 ± 0.41	**0.021**	[0.14 ; 1.715]
Difference in nb of M follicles	0.51 ± 2.91	-0.14 ± 2.85	0.65 ± 0.33	**0.052**	[-0.002 ;1.29]
Difference in nb of I follicles	0.25 ± 2.12	-0.06 ± 2.08	0.32 ± 0.24	0.191	[-0.16 ; 0.79]
Nb of oocytes	11.51 ± 10.34	11.34 ± 8.57	0.17 ± 1.10	0.514	[-1.99 ; 2.33]
Nb of MII oocytes	8.43 ± 8.66	8.99 ± 6.97	-0.56 ± 0.90	0.507	[-2.33 ; 1.22]
CCR n(%)	15 (13.2)	7 (3.1)		**< 0.001**	

Difference refers to the average of the differences of recruited follicles number between two ovaries for each patient in each group

*Abbreviations: Group S* Salpingectomy group (cases), *Group C* Control group, *CI* Confidence interval of the difference, *T* Total, *M* Mature, *I* Intermediate, *Nb* Number, *CCR* Cycle cancellation rate, *MII* Metaphase II

### Subgroup analysis

No statistically significant difference were found regarding any of the follicle size groups or oocyte yields when comparing either the salpingectomy for HSX subgroup to its respective control subgroup (Table 4), or the salpingectomy for EP subgroup to its control subgroup (Table 5). However, the CCR was statistically significantly increased in both subgroups S compared to their subgroups C (HSX group: 12.5% versus 4.4% respectively, p < 0.001; EP group: 8.8% versus 1.8% respectively, p = 0.004).

**Table 4:** Assessment of ovarian stimulation and oocytes retrieval outcomes in unilateral salpingectomy for hydrosalpinx and control subgroups

	Hsx Subroup S	Hsx Subroup C	Difference	*P*-value	95% CI
Difference in nb of T follicles	1.04 ± 4.81	0.04 ± 2.97	1.00 ± 0.60	0.1	[-0.19 ; 2.19]
Difference in nb of M follicles	0.75 ± 3.32	-0.1 ± 2.67	0.85 ± 0.48	0.08	[-0.09 ; 1.79]
Difference in nb of I follicles	0.29 ± 2.21	0.11 ± 2.13	0.18 ± 0.35	0.62	[-0.52 ; 0.88]
Nb of oocytes	12.47 ± 10.49	10.38 ± 7.35	2.09 ± 1.46	0.16	[-0.80 ; 4.97]
Nb of MII oocytes	9.18 ± 8.09	8.24 ± 5.94	0.94 ± 1.15	0.42	[-1.34 ; 3.22]
CCR n(%)	7 (12.5)	5 (4.4)		**< 0.001**	

Difference refers to the average of the differences of recruited follicles number between two ovaries for each patient in each subgroup

*Abbreviations: Group S* Salpingectomy group (cases), *Group C* Control group, *CI* Confidence interval of the difference, *T* Total, *M* Mature, *I* Intermediate, *Nb* Number, *CCR* Cycle cancellation rate, *MII* Metaphase II

**Table 5:** Assessment of ovarian stimulation and oocytes retrieval outcomes in salpingectomy for ectopic pregnancy and control groups

	EP Subgroup S	EP Subroup C	Difference	*P*-value	95% CI
Difference in nb of T follicles	0.42 ± 2.88	-0.39 ± 3.75	0.81 ± 0.57	0.16	[-0.31 ; 1.92]
Difference in nb of M follicles	0.23 ± 2.46	-0.18 ± 3.04	0.40 ± 0.47	0.39	[-0.51 ; 1.32]
Difference in nb of I follicles	0.19 ± 2.04	-0.21 ± 2.04	0.40 ± 0.33	0.23	[-0.25 ; 1.06]
Nb of oocytes	10.75 ± 10.27	12.30 ± 9.57	-1.55 ± 1.64	0.35	[-4.80 ; 1.69]
Nb of MII oocytes	7.81 ± 9.25	9.74 ± 7.82	-1.93 ± 1.39	0.17	[-4.68 ; 0.82]
CCR n(%)	5 (8.8)	2 (1.8)		**0.004**	

Difference refers to the average of the differences of recruited follicles number between two ovaries for each patient in each subgroup

*Abbreviations: Group S* Salpingectomy group (cases), *Group C* Control group, *CI*  Confidence interval of the difference, *T* Total, *M* Mature, *I* Intermediate, *Nb* Number, *CCR* Cycle cancellation rate, *MII* Metaphase II

## Discussion

In this retrospective study, we aimed to evaluate the impact of salpingectomy on the ovarian response in IVF cycles, in terms of follicular recruitment. Our case-control study was designed to compare two populations that were as similar as possible, thereby minimizing some of the biases of previous studies. The similar demographic characteristics of our two groups confirmed this matching, given that our patients had, as expected, equivalent AFCs and basal hormone levels, as well as their duration of infertility (Table 1). The only significant difference in demographic characteristics was in the type of infertility, with higher proportion of secondary and tubal infertility in Group S, which was consistent with the fact that half of these patients had undergone salpingectomy for EP. Likewise, it was also expected that there would be more cIVF in the study group, as most of these patients were suffering from female infertility (Table 2).

We observed that salpingectomized patients had a greater difference in T number of recruited follicles the day of trigger between their ovaries than that of their corresponding controls. Moreover, this greater difference was reflected by fewer T follicles being present on the salpingectomy side, supporting the hypothesis that the ovarian response to stimulation is impaired by this surgery. Despite the inter-ovarian differences in the number of both M and I recruited follicles being higher in Group S than in the Group C (Table 3), these differences failed to reach statistical significance for M follicles. However, the borderline statistical significance for those ≥ 16 mm in diameter follicles (*p* = 0.05) raises the possibility that significance may have been reached if a larger cohort of patients were studied. Moreover, a potential bias may have been introduced in the measurement of follicular diameters by ultrasound. Despite these measurements being performed in the same center by a small team of five people, all trained by the same senior practitioner and having homogeneous practices, the fact remains that a certain subjectivity persists in these measurements. Consequently, a 1-millimeter over- or under-estimation in diameter can result in a follicle falling into the mature or intermediate group; assessment of total follicles (≥ 13 mm) minimizes these measurement uncertainties providing the range in diameters is wide. The hypothesis of a negative impact of salpingectomy on follicular recruitment is also supported by our finding that an increased proportion of cycles were cancelled due to poor ovarian response in Group S compared to C, even though ovarian reserve measures, stimulation duration and total gonadotropin doses were equivalent between the two groups. However, despite higher cancellation rates and a larger inter-ovarian difference in total follicle number, the results of cycles leading to ovum pick-up showed an equivalent number of retrieved oocytes in Group S compared to their controls (Table 3). This observation may be due to a compensating phenomenon of the ovary contralateral to the salpingectomy, since it has previously been demonstrated that the response of one ovary adjusts to that of the other [20]. Whether there was truly no inter-ovarian difference in the number of mature oocytes between groups remains to be confirmed in a study investigating exclusively ICSI cycles, since there were significantly more cIVF cycles in Group S compared to Group C. In this fertilization technique, oocyte maturity is assessed on Day 1 after oocyte retrieval. In such cases, the cumulus-oocyte complexes, which are incubated for an additional 18 h in culture medium, may undergo in vitro maturation with extrusion of the first polar body.

Comparisons of the subgroups with their respective controls according to the indication of salpingectomy (HSX or EP), revealed no statistically significant inter-ovarian differences for the primary or most of the secondary outcomes. These findings may be due to a loss of statistical power resulting from the reduction in size of the compared cohorts, which were halved (57 patients in Subgroups S and 114 in their Subgroup C). However, two results remain notable: firstly, the CCR was still statistically significantly higher in both S subgroups compared to their respective subgroup controls (Tables 4 and 5). The second is that the difference of mature follicles were higher in the HSX subgroup comparing to EP subgroups with a non-significant but relatively small *p* = 0.08 (Tables 4 and 5).

Investigation with a larger number of patients in each subgroup and use of the same design as that of the present study is needed to further investigate whether the medical indication for salpingectomy modifies its impact on the ovarian response to hormonal stimulation. This is particularly since our previous study had suggested that salpingectomy for HSX was potentially more deleterious to the recruitment of M follicles, as its inflammatory component and its consequences on pelvic adhesions would likely make the resection procedure more delicate and potentially more damaging, even when not carried out in an emergency context as is the case for the EP indication [17]. This possibility could partly explain the contradictory conclusions of previous reports. In some studies including only patients who had undergone salpingectomy for EP, no difference in terms of follicular recruitment was found between the salpingectomy and control groups [20–23], or between the ipsilateral and contralateral ovaries when each patient served as her own control [24]. Whereas, when including patients who underwent salpingectomy exclusively for HSX, decreased numbers of T and M follicles on ovaries after salpingectomy were observed compared either with control patients [21] or those with a pre-surgery history [22]. Taken together, conclusions drawn from such studies remain open to criticism, whether due to the small size of cohorts, (for example, 15 [22] or 29 patients [25]) or because heterogeneous groups that were not matched on age or initial ovarian reserve were compared [21]. With the design of our current study, we have attempted to avoid these biases and so provide a more precise assessment of the impact of this surgery on follicular recruitment.

The hypothesis of damage to ovarian blood flow after salpingectomy has been advanced previously and some clinical observations support it. For instance, salpingectomized patients have onset of menopause earlier than controls after opportunistic salpingectomy in a retrospective study [26]. This trend of impairment of ovarian response after salpingectomy is also described in experimental studies where mouse ovaries after salpingectomy showed an increase in fibrosis and apoptosis scores and a decrease in secondary follicles, and in macroscopic cysts [27].

Furthermore, we can conclude that despite the divergence of studies carried out on this question, it is reasonable that resection and coagulation could have collateral damage on the ovarian vascularization due to the partially common ovarian and tubal anatomy. The extent of the damage will depend on the initial condition of the pelvis, the quality of the surgical procedure and the surgeon’s experience. Therefore, gynecological surgeons should be aware of the potential consequences of this surgery, which may seem quite trivial but which may have consequences on future fertility of the patient. Therefore, the management of infertile patients in ART centers diagnosed with hydrosalpinx should be considered. We recommend counseling on a case-by-case basis to discuss the benefit of postponing this surgery until after stimulation, oocyte retrieval and freeze-all. Regardless, care should be taken to minimize harm to the mesosalpinx.

The limitations of our study are its retrospective nature that involves potential missing data, and that it was not possible to distinguish between the number of oocytes obtained from each ovary : indeed, during the oocyte retrieval, follicular fluid is analyzed in by the biologist to isolate the oocyte without distinction between left and right ovary. Also, clinical outcomes such as implantation rates, pregnancy rates or live birth rates between the two groups were not compared. Furthermore, we lacked details regarding performance of the salpingectomy surgeries: Lamblin and colleagues showed that laparoscopic salpingectomy needs training, and junior internships have more efficacy, performance quality and speed of execution after several trials [28]. Also, vascular injuries may vary according to the instruments used for coagulation-section of the mesosalpinx as use of bipolar electric energy was associated with a decrease in AFC after salpingectomy, which was not observed with ultrasonic advanced energy (*Harmonic* ^®^) [29]. It would be interesting to match for experience of the surgeon (junior vs. senior) and for surgical devices (bipolar vs. ultrasonic advanced energy). It would be interesting to conduct a multicentric prospective randomized study on the salpingectomy technique before IVF, and to compare the number of oocytes retrieved from each ovary. Moreover, a multivariate model was not conducted as there was neither the power nor the number of subjects required to carry out a quality regression on the factors associated with the difference. In our opinion, the presence of a match associated with the small number of variables that could be entered into the model limits its construction. Residual confounding cannot be excluded, as multivariable adjustment or propensity score methods could not be reliably implemented given the limited sample size and number of events. This limitation should be considered when interpreting the results.

Despite the above limitations, our study has the strength of its design, in which we compared homogeneous groups and our sample size was relatively large compared with that in previous reports. In addition, its monocentric nature enabled us to ensure homogeneity in the practices of stimulation protocols and ultrasound follicle assessments. It would be interesting to validate the negative effect of salpingectomy on follicle recruitment with a future large prospective randomized control trial.

## Conclusion

Our findings indicate that salpingectomy has a negative impact on follicle recruitment in IVF cycles. This risk must be taken into consideration when scheduling this surgery for patients planning to undergo IVF treatment with prioritization given to oocyte retrieval with freeze-all. Surgeons should be aware of the need to preserve ovarian vascularity as far as possible.

## Data Availability

The datasets used and analysed during the current study are available from the corresponding author on reasonable request.
